# Eukaryotic Mitogenomes and Their Key Insights: Architecture, Evolution, and Mitonuclear Coevolution

**DOI:** 10.3390/ijms27209010

**Published:** 2026-10-11

**Authors:** Arpit Mehrotra, Abhilasha Sood, Yuri P. Kartavtsev

**Affiliations:** 1 Department of Allied Health Sciences, Chitkara School of Health Sciences, Chitkara University, Punjab 140401, India; arpit.mehrotra@chitkara.edu.in (A.M.);; 2 A.V. Zhirmunsky National Scientific Center of Marine Biology, Far Eastern Branch, Russian Academy of Sciences, Vladivostok 690041, Russia

**Keywords:** bioenergetics, mitogenomes, mitogenome architecture, mitochondrial evolution, mitonuclear coevolution, heteroplasmy, phylogenomics, mitochondrial disease, Dn/Ds, substitution rates for non-synonymous and synonymous mutations (substitutions), nDNA, nuclear DNA, mtDNA, mitochondrial DNA, PCGs, protein-coding genes

## Abstract

Mitochondrial genomes (mitogenomes) are no longer considered entities solely responsible for cellular bioenergetics, but as dynamic, multifaceted, and synchronized systems that integrate genome architecture, evolutionary processes, and organismal physiology. The present review summarizes the contemporary understanding of mitogenome organization and its diversity, evolution, and associated functions across eukaryotic life, with emphasis on mitonuclear co-evolution and relevance to human health. Moreover, the conserved and lineage-specific characteristics of mitogenome architecture, including its genetic content, topology, and regulatory elements, and their roles in governing the precise replication and transcription of the mitogenome are also comprehensively discussed. Additionally, the review explores the role of evolutionary dynamics, with emphasis on mutation-rate heterogeneity, recombination dynamics, gene rearrangements, and the interaction between mutation, natural selection, and genetic drift in shaping mitochondrial genome diversity and evolutionary trajectories. Comparative approaches have revealed extensive diversity in mitogenome structural organization and composition, which are closely associated with life-history traits of organisms, including metabolic rate, longevity, and reproductive characteristics. Notably, at the functional level, the variability potential of a mitogenome significantly influences oxidative phosphorylation efficiency, reactive oxygen species generation, and the orchestration of cellular signaling cascades. Fundamental to these processes is the existence of coordinated mitonuclear interrelationships, wherein coevolution between mitochondrial and nuclear-encoded genes safeguards bioenergetic functional integrity, whereas disruptions in this coordination may lead to reduced cellular fitness and progression of several diseases. Particularly in humans, mutations in mitochondrial DNA (mtDNA) and heteroplasmy significantly contribute to the development of primary mitochondrial disorders and are increasingly implicated in other complex disease etiologies, including neurodegeneration, metabolic syndromes, and cancer. The paper also summarizes the phylogenetic utility of mitogenomes in the genomic era, accentuating the need to employ integrative approaches that combine mitochondrial and nuclear data. Lastly, the role of emerging technological platforms, including long-read sequencing and multi-omics, is also discussed, since these approaches are expected to transform the current landscape of mitogenome research. Altogether, the manuscript positions mitogenomes as indispensable entities bridging the gaps between evolutionary histories, physiological functions, and personalized precision medicine.

## 1. Introduction

**Figure 1:**
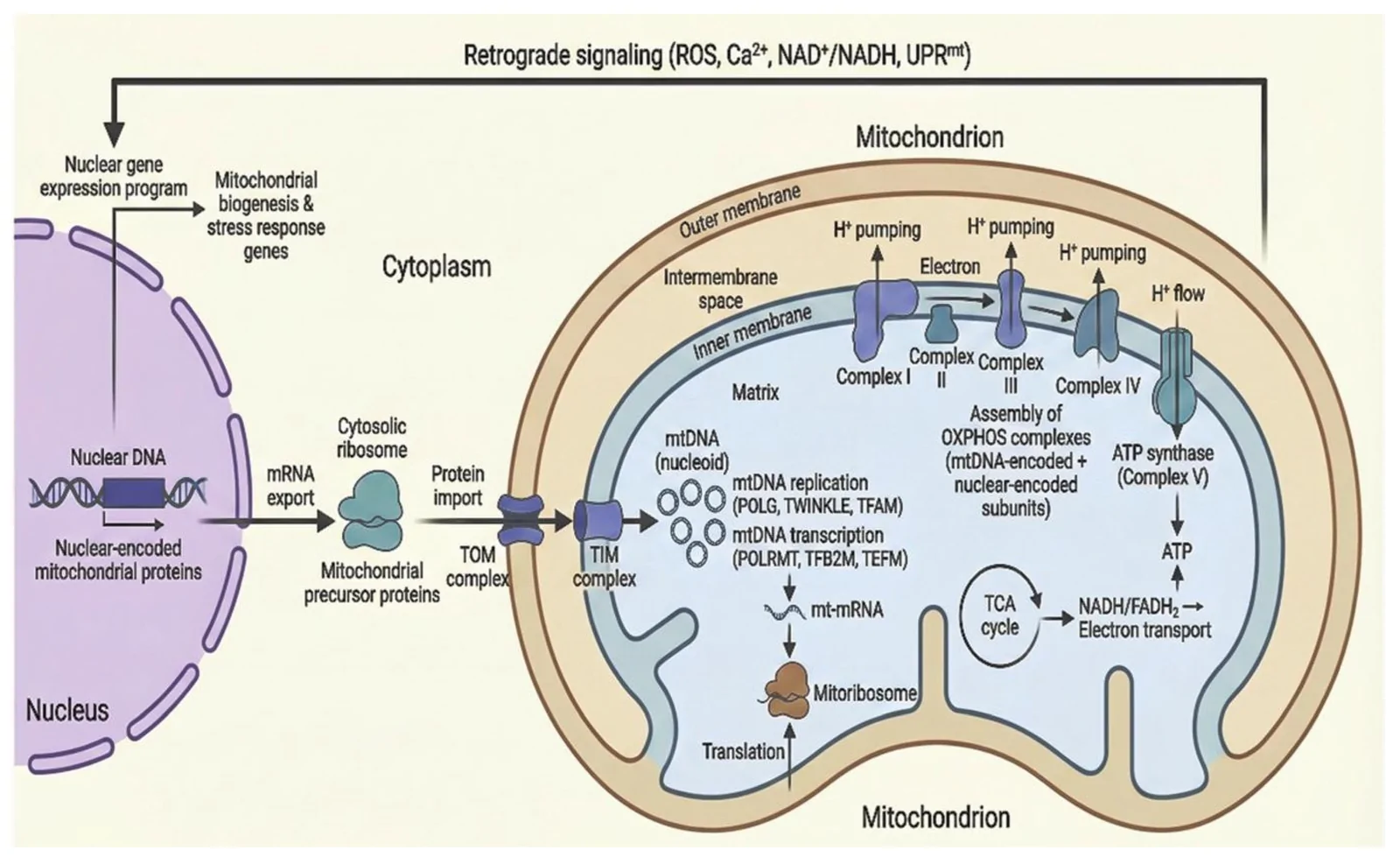
The mitogenome as a multidimensional system. Schematic representation of mitochondrial genome architecture, including protein-coding genes (PCGs), rRNAs, tRNAs, and regulatory elements controlling replication and transcription. UPR^mt^ (mitochondrial unfolded protein response) is a cellular stress response related to the mitochondria. TIM/TOM denotes a protein complex in cellular biochemistry. Mitochondrial PCGs encode core subunits (proteins/polypeptides) of oxidative phosphorylation (OXPHOS) complexes, whereas the majority of mitochondrial proteins are encoded by the nuclear genome and imported into the organelle. Coordinated expression of mitochondrial and nuclear genes enables assembly of respiratory-chain complexes, ATP synthesis, and cellular metabolic regulation. The figure highlights the interconnected processes of replication, transcription, translation, and mitonuclear communication that collectively underpin mitochondrial function, evolutionary dynamics, and organismal physiology. The Figure 1 image is an original that was made by A. Mehrotra using BioRender software (https://www.biorender.com/).

Mitochondrial DNA (mtDNA) has recently been recognized as a keystone hub in modern genetics that surpasses its classical role in modulating cellular energy metabolism. Formerly considered a highly compact and static remnant of an ancestral endosymbiont, mtDNA is now recognized as an extremely dynamic genomic entity with significant implications for evolutionary biology and the regulation of cellular physiology, including direct involvement in the maintenance of human health [1,2]. This reassessment has been made largely possible by recent advancements in long-read sequencing technology, single-cell analyses, and integrative genomic approaches, which have revealed unexpected structural diversity, variable patterns of inheritance, and ubiquitous mitonuclear communications across eukaryotes [3,4]. Although the canonical animal mitogenome is compact (circular and approximately 16–20 kbp in size) and encodes for 13 oxidative phosphorylation (OXPHOS) proteins, 22 tRNAs, and 2 rRNAs, the magnitude of structural variation across different taxa is remarkable [5,6]. Unusually, plants and protists do not follow the established paradigm of organellar streamlining by recurrently exhibiting massively expanded mtDNAs with introns and with some plant mtDNAs exceeding 11 Mb in size. Such gigantic, multifaceted genomes are driven by low substitution rates, higher recombining repeats, mobile genetic elements, and frequent mitochondrion-to-mitochondrion horizontal gene transfer, thereby posing a challenge to the traditional paradigm of organellar functioning [7]. Even among metazoans, genomic rearrangements, control region duplications, and gene content shifts are common, often driving adaptation by altering metabolic rates, thermal tolerance, and lifespan [8]. Moreover, the mitogenome is a dynamic, multidimensional system integrating structural organization, replication, and transcription within the cellular environment (Figure 1). The circular mitogenome encodes vital components for oxidative phosphorylation, while its maintenance and expression depend significantly on nuclear-encoded proteins, including polymerase gamma (POLG), mitochondrial transcription factor A (TFAM), mitochondrial RNA polymerase (POLRMT), mitochondrial single-stranded DNA-binding protein (mtSSB), transcription factors B1 and B2 (TFB1M/TFB2M), Twinkle DNA helicase, and mitochondrial ribosomal proteins (MRPs). These nuclear-imported proteins, along with others involved in mtDNA repair and RNA processing (such as TEFM and RNase P), are considered fundamental for the proper functioning of the 13 polypeptides encoded by the mitogenome. Although mitochondria retain a compact genome, its replication, transcription, RNA processing and translation depend extensively on nuclear-encoded factors imported into the organelle, highlighting the functional interdependence of the mitochondrial and nuclear genomes. This functional dependence extends from basic genome maintenance and expression to the assembly and regulation of mitochondrial pathways [9,10]. Additionally, the replication and transcription processes occur in close coordination, being regulated by transcription factors (TFAM, TFB1M/TFB2M) imported from the nucleus into mitochondria. Figure 1 highlights the bidirectional exchange of signals and proteins between mitochondrial and nuclear compartments, thereby emphasizing functional interdependence. Such mitonuclear coordination ensures mitochondrial integrity, bioenergetic efficiency, and adaptive responses to metabolic demands, positioning the mitogenome as a central hub in maintaining cellular homeostasis and cross-compartmental communications. Such mitonuclear coordination links mitochondrial genome variation to cellular metabolism, physiological adaptation, and organismal homeostasis.

The evolutionary dynamics of the mitogenome or mtDNA have also been driven by its unique replication and maternal inheritance [11,12]. Uniparental (typically maternal, but see some exceptions below) transmission and the absence of conventional recombination diminishes effective population size (Ne) by at least one-quarter [13,14], with a concomitant increase in the influence of genetic drift, which altogether augments lineage sorting relative to nuclear loci [15]. Nevertheless, this diminished operational population size simultaneously exaggerates the stepwise accumulation of deleterious mutations (phenomenon theoretically referred as Muller’s ratchet) [16], unless neutralized via mitochondrial genetic bottleneck or selection against dysfunctional variants during the germline transmission process [17]. Recently, the role of mitonuclear coevolution was highlighted by showing that rapid changes in mtDNA mutation rate augment compensatory evolution in nuclear-encoded mitochondrial (N-mt) proteins. Since mtDNA and nuclear DNA evolve independently, coadaptation is vital, and compensatory changes in both genomes ensure that translated proteins match structurally, thereby sustaining efficient energy production while alleviating proteotoxic stress and reactive oxygen species (ROS) leakage [18]. Such coadaptation confirms that the protein subunits from both genomes must fit together “like a lock and key” to function efficiently [19]. Disruption of mitonuclear compatibility can consequently influence bioenergetic functions, reproductive fitness, adaptation, and disease susceptibility [20,21].

The recent conceptual shift from viewing the mitogenome merely as a static, maternally inherited marker of genetic disease in humans to recognizing it as an interactive whole-genome hub has reshaped investigators’ understanding of mitochondrial diseases [22,23]. Moreover, high-depth sequencing approaches have revealed extensive heteroplasmy, wherein mixtures of wild-type and mutant mtDNA generate tissue-specific threshold effects that contribute to phenotypic variability, as evidenced by review data available from several organisms investigated [24,25]. Evidently, minute discordance in mitonuclear functioning has been increasingly implicated in age-related pathologies, neurodegenerative diseases, and complex metabolic traits, thereby expanding the clinical relevance of human mitogenomes beyond classical mitochondrial disorders [26]. Despite substantial advances in mitochondrial genetics, mitogenome architecture, evolutionary dynamics, physiological function, and clinical consequences have often been investigated as partially separate fields. This separation can obscure the biological continuum through which mitochondrial genome variation interacts with the nuclear genome and environmental context to influence cellular metabolism, organismal performance, life-history strategies, and disease susceptibility [27]. Therefore, this manuscript integrates these perspectives to examine how mitogenome architecture and evolutionary history shape mitochondrial dynamics, how mitonuclear coevolution constrains or facilitates adaptation, and how these processes ultimately manifest at organismal levels.

Unlike earlier published reviews that have predominantly focused on mitochondrial genome evolution, mitonuclear interactions, mitochondrial disease or phylogenetic applications individually, the present overview connects these domains within a unified evolutionary framework spanning diverse eukaryotic lineages [28,29]. The paper further considers emerging long-read sequencing, phylogenomics and multi-omics approaches, and identifies unresolved questions concerning the relative contributions of mutation, drift, and selection. Furthermore, the consequences of mitogenomic structural innovation, mitonuclear incompatibility, and the potential of mitochondrial variation to inform evolutionary and precision medicine have also been considered in drafting of the paper. Thus, by connecting genome architecture and evolutionary history with physiological function and clinical phenotype, the presented review warrants further investigations that could explicitly connect the missing links and highlights opportunities for comparative and mechanistic and translational research.

## 2. Architecture of Mitogenomes

**Figure 2:**
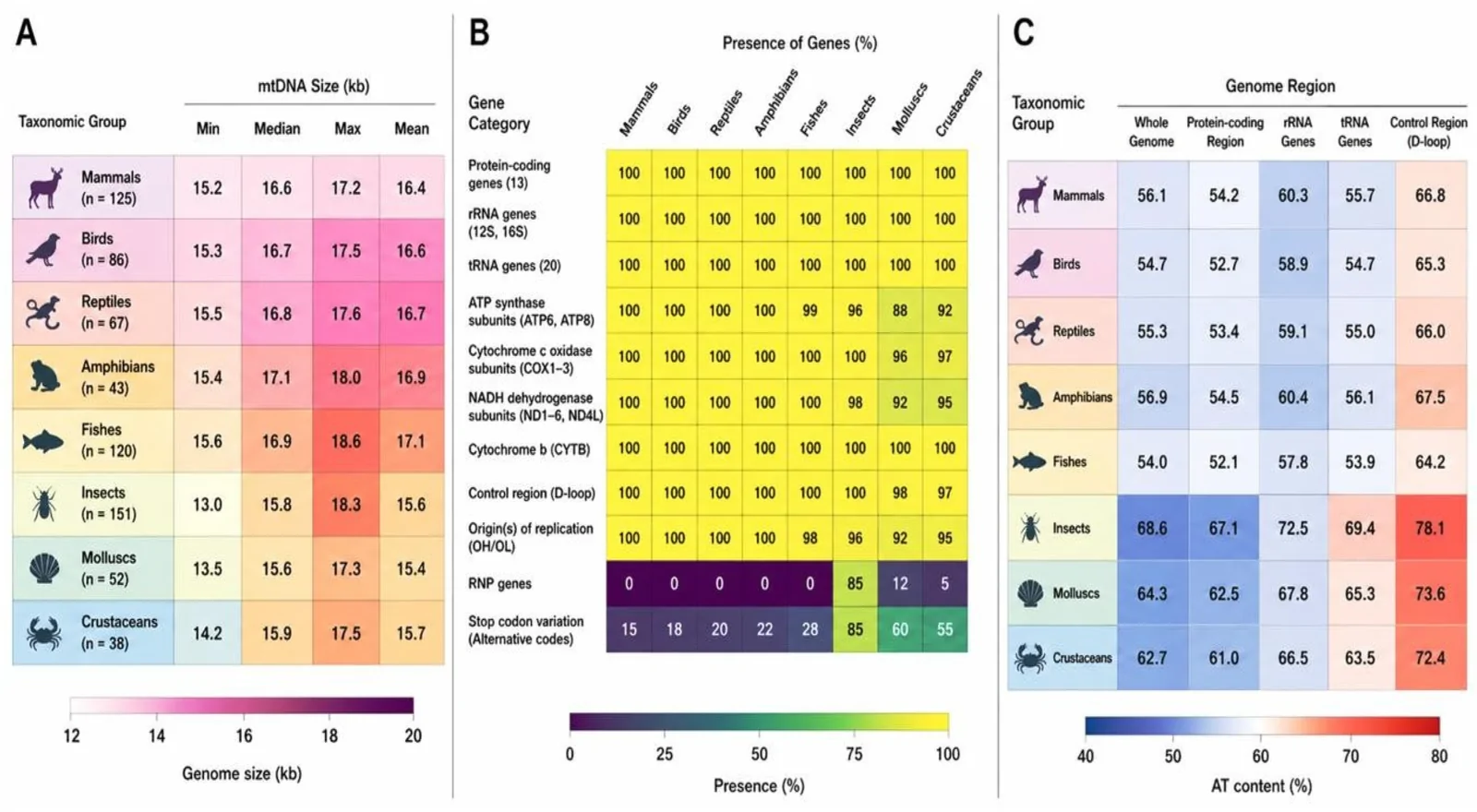
Comparative structural features of mitochondrial genomes across major eukaryotic lineages. (**A**) Heatmap summarizing mitochondrial genome size (in kb) across taxonomic groups, including mammals, birds, reptiles, amphibians, fishes, insects, mollusks, and crustaceans. Values represent minimum, median, maximum, and mean genome sizes, highlighting lineage-specific variation and relative compactness of metazoan mitogenomes. (**B**) Heatmap depicting the presence (%) of key mitochondrial gene categories across taxa. Conserved gene sets include protein-coding genes (PCGs), rRNAs, and tRNAs, whereas variability is observed in ATP synthase subunits, NADH dehydrogenase genes, control regions and origins of replication, D-loops or control regions (CRs). The panel also shows lineage-specific features such as RNA-processing genes and variation in stop codon usage. (**C**) Heatmap showing AT content (%) across different mitochondrial genome regions: whole genome, protein-coding regions, rRNA genes, tRNA genes, and CRs. The facts reveal compositional biases among taxa, with generally higher AT content in invertebrates and in regulatory regions such as the control region. Heatmaps represent a synthesis of general patterns reported across comparative mitochondrial genomics studies and are intended to provide a conceptual overview of lineage-specific variation rather than a comprehensive quantitative meta-analysis. Data were compiled from representative comparative studies of mitochondrial genome architecture across eukaryotes [45,46,47,48,49,50].

Mitochondrial genomes demonstrate architectural diversity that contradicts their relatively small size in several biological lineages and are extraordinary variable in plants and ancient metazoans. Classical depictions of animal mtDNA feature a compact, circular, double-stranded molecule (~14–20 kb) encoding a conserved set of 37 genes. However, comparative genomics and modern sequencing techniques reveal a spectrum of organizational variants shaped by lineage-specific evolutionary pressures [5,30], as illustrated in Figure 2. This architectural variability highlights differences in replication strategies, recombination dynamics, genomic stability, and interactions with the nuclear system or other cellular elements, thereby accentuating the need to consider mitogenomes as adaptable genomic architectures of cells and entire organisms, rather than mere static organellar entities [31]. In metazoans, the canonical animal mitogenome encodes 13 PCGs involved in OXPHOS functioning, two ribosomal RNAs, and usually 22–24 tRNAs, which are most variable in number and specific set. It includes a non-coding control region (the D-loop in mammals or the A+T-rich region in invertebrates) that coordinates replication, the origin of heavy/light (or +/−) strand synthesis, and transcription (promoter) mechanisms [32]. Despite broad conservation of gene content, the gene order (synteny) is surprisingly fluid. For instance, extensive rearrangements of tRNAs and even protein-coding genes have been demonstrated across *Arthropods* (insects and crustaceans), nematodes, and bivalve mollusks, which are frequently found to be associated with lineage-specific life-history traits and metabolic demands [33,34,35,36]. Such rearrangements challenge the classical hypothesis of mtDNA as a rigidly conserved marker for phylogenetics and necessitate careful investigation of gene synteny when interpreting evolutionary histories to circumvent imprecise phylogenetic interpretations [37,38].

As discussed above, a contrasting pattern has been demonstrated to occur in plants and protists, wherein the size of the mitogenome and its architectural organization are far more heterogeneous in nature. This variation extends from relatively compact genomes to exceptionally large genomes exceeding several megabases (Mb). For instance, the mitogenome of the plant *Silene conica* approaches 11 Mb, whereas other angiosperms possess substantially smaller mitochondrial genomes, illustrating that genome size is poorly predicted by mitochondrial gene number alone. In addition to size variation, plant mitogenomes can contain extensive repeated sequences, foreign DNA derived from plastids, the nucleus, introns, and alternative genomic configurations generated through recombination. In some species, mitochondrial DNA consequently occurs as a heterogeneous population of circular, linear, and branched molecules rather than as a single uniform chromosome [39,40]. Moreover, repeated sequences provide substrates for homologous recombination and can contribute to the generation of alternative genome configurations, multipartite structures, and sub-stoichiometric forms within plant mitochondria [41,42]. Moreover, extensive RNA editing is widespread in plant mitochondria and can further complicate genotype-to-phenotype relationships by modifying mitochondrial transcripts post-transcriptionally. This process encompasses the conversion of cytidine to uridine at hundreds of specific sites within mitochondrial transcripts [43,44].

Among the protists, mitogenome configurations range from linear chromosomes with telomere-like ends to extremely fragmented minicircles, as observed in kinetoplastids, wherein hundreds of circular molecules encode a mosaic of genes and guide RNAs. In kinetoplastids, these architectures are supported by specialized replication and segregation mechanisms associated with maxicircle–minicircle systems, which differ substantially from the organization of canonical metazoan mitogenomes [51,52]. However, Jakobids retain comparatively gene-rich mitogenomes that preserve several ancestral features [53,54], whereas Apicomplexans possess highly reduced mitogenomes [55]. Other protist lineages possess linear, fragmented, or highly rearranged mitogenomes, demonstrating that mitochondrial genetic information can be partitioned among structurally distinct molecules [56,57]. These examples emphasize that protist mitogenomes cannot be adequately represented by the compact circular model derived from animals and instead constitute a broad spectrum of genomic reduction, fragmentation, and a molecular specialization. Thus, the contrasting architectures observed across eukaryotes suggest that mitogenome evolution has followed multiple lineage-specific trajectories rather than a single model of genome organization [58,59,60].

In animals, strong functional constraints and genome compaction have generally maintained small, gene-dense mitogenomes, although gene-order rearrangements have been shown to exist in particular lineages [61]. By contrast, plant mitogenomes frequently undergo extensive expansion and structural remodeling associated with repetitive sequences, recombination, and intracellular DNA transfer [62]. Fungal mitogenomes occupy an additional evolutionary spectrum, with size and organization influenced by introns, mobile genetic elements, recombination, and lineage-specific gene loss [63]. Protists exhibit some of the extreme forms of mitochondrial genome reduction, fragmentation, and structural innovations, including minicircle–maxicircle systems and extensive RNA editing [64]. These differences suggest that mitogenome architecture reflects the interaction of functional constraint with lineage-specific rates of mutation, recombination, DNA repair, gene transfer, and transmission. Thus, genome size, topology and gene organization should not be interpreted as isolated structural traits, but as outcomes of distinct evolutionary regimes operating within different cellular and ecological contexts.

Structural diversity in mitochondrial genomes is further exemplified by doubly uniparental inheritance (DUI), an unusual transmission system that has been discovered in several bivalve lineages, including *Mytilidae*, *Unionidae*, *Mactridae* and *Veneridae*, wherein distinct female (F-type) and male (M-type) mitochondrial genomes are inherited through maternal and paternal lineages, respectively [65,66,67,68]. Unlike the predominantly maternal transmission observed in many metazoans, DUI produces distinct female and male transmitted mitochondrial genomes in several bivalve lineages, with substantial variation in their sequence divergence, gene organization, and evolutionary trajectories [69,70,71]. On the other hand, cnidarians and sponges possess linear mitogenomes with telomeric repeats, demonstrating adaptations to specific cellular microenvironments or life histories [72]. The CRs in mtDNA in vertebrates show structural plasticity within largely conserved genomic content. Variations in length of mitochondrial control-region and sequence composition have been investigated in relation to replication, transcription, and organismal traits, including metabolic and life-history variables; however, the consistency and evolutionary basis of these associations across vertebrate lineages remain unresolved [73,74].

Additionally, tandem repeat expansions within control regions also serve as hotspots for slipped-strand replication, which may encourage heteroplasmy dynamics in mitochondrial populations. At the molecular level, mitogenome architecture can be influencing by lineage-specific differences in DNA replication, repair, and recombination, as well as repeat abundance, intracellular DNA transfer and genome transmission [75]. In plants, for example, MSH1 and recombination-associated pathways contribute to the maintenance and restructuring of repeat-rich mitochondrial genomes, whereas the relative contributions of analogous mechanisms vary across eukaryotic lineages. The extent to which individual nuclear-encoded maintenance factors determine lineage-specific architectural evolution remains an active area of investigation. Nuclear-encoded factors such as TWINKLE helicase, DNA polymerase-gamma (POLγ), and mitochondrial single-stranded DNA-binding proteins (mtSSB) modulate replication fidelity and structural stability, linking mitogenome organization directly to nuclear genetic control. Altogether, the architectural diversity of mitogenomes reflects a balance between functional constraint and evolutionary flexibility [76,77,78,79]. By contextualizing these architectures across lineages, investigators are expected to decipher how architectural variation interfaces with metabolic demands, population genetic processes, and ecological adaptation.

## 3. Evolutionary Dynamics of Mitochondrial Genomes

The mitogenome presents a distinctive and complex system of network relations and structural units that distinguish its dynamic functioning from those of nuclear machinery. The complex interplay of mutation pressure, selection, genetic drift, and demographic history shapes variation in substitution rates, structural features, and functional constraints, with consequences for fundamental aspects of biology and genetics, including organismal adaptation, evolution, phylogenetic inference, disease susceptibility, etc. [80,81]. Mitochondrial evolutionary dynamics are therefore best understood as the outcome of a shifting balance among mutation, selection, and genetic drift rather than the consequence of any single dominant force. Mutation supplies genetic variation, whereas selection determines the persistence or removal of variants according to their effects on mitochondrial function and organismal fitness [82]. Genetic drift can become particularly influential when mitochondrial effective population size (Ne) is reduced, but its magnitude depends on inheritance mode, reproductive biology, demographic history and the extent of mitochondrial recombination [83]. Consequently, the same mitochondrial variant may experience different evolutionary trajectories in different lineages. Strong purifying selection can constrain variation in functionally essential OXPHOS genes, whereas relaxed selection, demographic bottlenecks or small effective population size may permit mildly deleterious variants to persist [84]. Conversely, recurrent positive or balancing selection may promote the retention of variants associated with adaptation to particular ecological or physiological environment. These processes can operate simultaneously and their relative importance is expected to vary across taxa, tissues and evolutionary timescales [85]. Therefore, understanding for peculiarities of evolutionary dynamics in an organism lineage necessitates incorporating classical population genetic knowledge with emerging insights from the approaches of modern comparative genomics, high-throughput sequencing technologies and experimental research on evolution in peculiar species and organisms.

A major determinant of mitochondrial population genetics in many metazoans is predominantly uniparental, usually maternal inheritance, which generally results in a smaller Ne for mitochondrial genomes than for autosomal nuclear loci [86,87,88]. However, the magnitude and evolutionary consequences of this reduction depend on sex-specific demography, reproductive variance, inheritance patterns and population history. A reduced Ne can increase the relative increase of genetic drift and thereby facilitate the persistence and, in some demographic contexts, fixation of mildly deleterious mitochondrial variants [89,90,91]. However, reduced Ne does not imply that drift invariably dominates mitochondrial evolution. The fate of a mitochondrial variant reflects the interaction between its selection coefficient, mutation rate, demographic history and transmission dynamics. Strong purifying selection can efficiently remove functionally deleterious variants, whereas population bottlenecks, reproductive skew or relaxed selection can increase their probability of persistence [92]. Thus, mutation, selection and drift should be considered interacting rather than competing explanations for mitochondrial variation. Evidence for the accumulation or elevated fixation of mildly deleterious mitochondrial variants has also been reported in metazoans, consistent with the potential influence of reduced Ne and limited recombination [93]. However, analogous processes in plants may operate under different genomic and inheritance regimes, with consequences often expressed through cytonuclear interactions, reproductive traits and fitness rather than through the same aging-related phenotypes observed in animals [94]. Nevertheless, strong purifying selection on core OXPHOS genes remains universal, as these proteins interface with nuclear-encoded subunits, which are essential for regular bioenergetic functioning. The critical nature of such “mitonuclear” interaction causes intense selective pressure that eliminates mutations causing dysfunctions [95,96], which will be further discussed in a later section of the manuscript.

Mitochondrial mutation rates demonstrate striking variability among different taxa. For example, in vertebrates, mtDNA evolves rapidly, frequently with an order of magnitude faster than nuclear DNA, thus making it exceedingly useful for resolving recent divergences [97,98,99]. However, in plants and some protists, mitochondrial mutation rates are paradoxically lower at nucleotide level despite large and structurally dynamic genomes [100]. This decoupling of mutation rate and mitochondrial genome architecture shapes differences in DNA repair efficiency, including its replication fidelity across lineages. For example, plant mitochondria exhibit high rates of recombination and architectural rearrangements facilitated by the presence of repeated elements, though also preserve low substitution rates due to competent error correction mechanisms [101,102]. This existence of substitution rate heterogeneity within metazoans correlates well with life-history traits. For instance, short-lived species with high metabolic demand tend to accrue substitutions more rapidly, which is consistent with the metabolic rate hypothesis [103,104,105]. This suggests that enhanced oxidative stress and replication turnover increases mutational input [106,107]. On the other hand, long-lived species with slower metabolic rates frequently demonstrate lower mtDNA substitution rates, possibly reflecting stronger purifying selection or reduced replication load [108]. These associations, while vigorous in broad patterns, are not universal and therefore may differ with ecology and Ne impact [109,110], thus emphasizing the requirement for lineage-specific analyses. Recombination in mitochondrial genomes has long been under intense debate, often considered rare or absent due to stringent uniparental inheritance and the resulting homoplasmic state (signifying identical mtDNA molecules within a cell). Animal mtDNA is conventionally considered non-recombining; however, investigations from high-depth sequencing approaches (PacBio^®^ HiFi or high-coverage next-generation sequencing) discloses rare but detectable recombination events in humans and other vertebrates [111,112]. In contrast, recombination is widespread and often an important component of mitochondrial genome maintenance and structural variation in plants and fungi, where repeated sequences and recombination can generate alternative genomic configurations and sub-stoichiometric forms [42,113]. Collectively, mitochondrial evolutionary dynamics cannot be reduced to a single model of mutation accumulation or drift. Instead, mitogenomes occupy different evolutionary regimes in which mutation, selection and drift interact with inheritance mode, recombination, demographic history, life-history strategy and mitonuclear compatibility [114].

In summary, predominantly maternally inherited metazoan genomes, reduced Ne and limited recombination may increase the relative influence of drift, although strong purifying selection continues to constrain functionally important variants [115]. Plant and fungal mitogenomes, by contrast, experience extensive recombination and structural turnover, whereas highly divergent protist systems demonstrate additional trajectories involving genome reduction, fragmentation and specialized inheritance or replication [63,116]. These studies emphasize that mitochondrial evolutionary dynamics are fundamentally lineage-dependent and that conclusions derived from one taxonomic group should not be automatically extrapolated across all eukaryotes, since such lineage dependence may support mitonuclear discordance, particularly in circumstances wherein introgression, incomplete lineage sorting or demographic asymmetry causes mitochondrial and nuclear genomes to retain different evolutionary histories [117]. Therefore, an integrative framework incorporating population genetics, genome architecture, life history and cellular function is warranted to explain the heterogeneous evolutionary trajectories of mitogenomes (Figure 2).

## 4. Mitogenome Diversity and Life-History Strategies

Mitogenome diversity is not an isolated phenomenon in organismal biology. Rather, it is deeply intertwined with the life history of each organism, heredity patterns and species evolution. Variation in metabolic rate (energy expenditure per unit body mass), lifespan, and reproductive strategy and Ne size largely influences the mutation rate, the severity of selective constraints and the cumulative structural organization of mitogenome [106,113]. Therefore, understanding such relationships could provide a holistic framework that might be useful for interpreting both macro-evolutionary patterns and lineage-specific differentiation peculiarities. Among these relationships, one of the most comprehensively examined relationships is that between mitochondrial mutation rate and metabolic rate. The metabolic rate hypothesis proposes that increased metabolic activity may influence mitochondrial mutational input through greater oxidative stress and/or increased mitochondrial replication and turnover [106]. This relationship is not necessarily direct, because mutation accumulation also depends on replication fidelity, DNA repair, mitochondrial quality control, and the efficiency of selection against deleterious variants [118]. Thus, comparative studies across vertebrates and insects have reported associations between mass-specific metabolic rate and mitochondrial substitution rates, particularly in some small-bodied mammals and flying insects [119,120]. However, the strength and direction of these associations vary among lineages, indicating that metabolic intensity alone is insufficient to predict mitochondrial evolutionary rate. Recent evidence further suggests that replication-associated errors during mtDNA turnover and maintenance may contribute more substantially to heritable mitochondrial mutation than previously assumed, challenging a simple model in which oxidative damage is the primary source of mitogenome mutations [121,122,123,124]. Thus, although ROS can influence mitochondrial DNA integrity, mutation accumulation is likely to reflect the combined effects of polymerase fidelity, replication dynamics, DNA repair, oxidative damage and selective filtering across generations [125]. These findings highlight the significance of replication dynamics and germline blockages in determining subsequent evolutionary rates.

Long-lived species frequently exhibit reduced mitochondrial substitution rates and stronger signatures of purifying selection, but this pattern is not universal and appears to be strongly lineage-dependent [108]. Birds and bats are a particularly informative exception. Despite relatively high metabolic demands, several long-lived species exhibit slower-than-expected mitochondrial evolutionary rates, suggesting that metabolic intensity alone cannot explain variation in mitochondrial mutation or substitution rates [126,127,128]. These observations point towards additional influences, including DNA repair capacity, mitochondrial quality control, germline selection, generation time, and demographic history [129]. They indicate that metabolic rate alone cannot explain heterogeneity in mitogenomic evolutionary rates: instead, life history trade-offs involving reproduction, somatic maintenance, longevity, generation time and mitochondrial quality control should be incorporated into comparative evolutionary models. Moreover, the Ne variation is well known to exercise a dominant influence on mitogenome diversity despite some complications [130,131]. Since the mitogenome is characteristically haploid and maternally inherited, its Ne is approximately 1/4–1/2 that of autosomal nuclear loci under equal sex ratios [131,132]. However, this theoretical reduction in Ne should not be interpreted as a fixed property of all mitochondrial populations, because sex ratio, variance in reproductive success, population structure, inheritance mode, demographic history, and occasional recombination can substantially modify realized mitochondrial Ne [133]. Reduced Ne augments genetic drift, thereby increasing the probability of fixation of slightly deleterious mutations and accelerating lineage sorting, as demonstrated by simulations and supported by empirical data from the literature [134].

Comparative genomic studies have reported associations between smaller effective population sizes and elevated non-synonymous divergence in some taxa, consistent with relaxed purifying selection [134,135]. Nevertheless, the Dn/Ds ratio is influenced by several factors, including demographic history, selection, mutation rate variation, and gene-specific functional constraint, and therefore should not be interpreted as a direct proxy for Ne only or as natural selection at gene markers [136]. Conversely, species with large Ne may maintain stronger selective constraints and lower non-synonymous divergence in comparison to species with small Ne, which are more susceptible to genetic drift [135]. Reproductive and biochemical adaptive strategy may also may modulate mitogenomic dynamics [137,138]. In particular, in taxa with skewed sex ratios or complex inheritance systems (as exemplified with DUI, in bivalves), selection may act differently on male- and female-transmitted mitogenomes, producing divergent evolutionary trajectories within a single species [6,9,10,11,43]. Similarly, species experiencing persistent population bottlenecks may display rapid shifts in haplotype frequencies due to drift, rather than adaptive change [10].

Evidently, the association between life-history traits and mitochondrial evolution is not fully predictive. Ecological specialization, thermal adaptation, and migratory behavior can modulate metabolic demand and selective pressures on mtDNA, independently of body size or lifespan [139]. Although slow life histories, characterized by long lifespans and large body size, are conventionally associated with slower rates of mitochondrial evolution, these other biological and ecological factors may also significantly contribute to complex pattern of variation and divergent evolutionary outcomes [139,140,141]. Relationships among metabolic rate, longevity and mitochondrial evolutionary rate are probabilistic rather than deterministic, because mutation input and the subsequent fate of mitochondrial variants are jointly influenced by replication fidelity, DNA repair, generation time, effective population size, reproductive strategy, germline selection, ecological conditions, and mitonuclear interactions [142]. The relationship between life history and mitogenomic evolution is therefore inherently multifactorial. Metabolic rate may influence mitochondrial turnover and mutational input, whereas longevity can impose stronger selection for mitochondrial maintenance and quality control. However, these effects are mediated by generation time, reproductive strategy, effective population size, demographic history, DNA repair, germline selection, ecological conditions, and mitonuclear interactions [143]. Consequently, species with similar metabolic rates or lifespans may exhibit markedly different mitochondrial evolutionary rates, while lineages with contrasting metabolic demands can converge on similar substitution patterns [109]. Mitogenomic diversity should therefore be interpreted as the outcome of interacting physiological, demographic and evolutionary processes rather than as a simple consequence of metabolic intensity or longevity. Integrating these variables provides a more robust framework for distinguishing adaptive signals from demographic and mutational effects in comparative analyses.

## 5. Functional Implications of Mitogenomic Variation

Variation within mitogenomes can have significant functional consequences for cellular physiology and organismal fitness. Although the mitogenome combines a comparatively small set of genes, these genes play essential role in oxidative phosphorylation, which mediates the generation of ATP in eukaryotic cells (Table 1). Subsequently, even marginal variation in mitochondrial sequence or copy number can greatly influence metabolic performance, redox balance and life-history traits in organisms [144,145].

Table 1 summarizes the relationships between key types of mitogenomic variations and their associated functional and clinical consequences in humans. It highlights that characteristic features of mitogenomic variations (e.g., heteroplasmy, elevated mutation rates in the mitogenome, deletions, and mitonuclear incompatibility) all converge to impair OXPHOS, thereby disrupting cellular energy metabolism. Such molecular defects translate into different phenotypes, ranging from classical mitochondrial disorders to complex diseases, including neurodegeneration, cardiometabolic dysfunction and aging-related decline. Notably, the table also underscores the evidence-dependent nature of mitogenomic effects, wherein thresholds of the mutation load in the mitogenome and its interaction with the nuclear genome critically determine disease manifestation. Data presented in Table 1 also highlight the translational opportunities, including the usage of mtDNA variants as biomarkers, and the development of targeted therapeutic approaches.

At the molecular organization level, mtDNA encodes core subunits of mitochondrial respiratory chain complexes I, III, IV, and V, all of which function in close structural association with nuclear-encoded proteins. Amino acid substitutions or changes in mitochondrial polypeptide sequences coded by mitogenomes can therefore alter electron transport chain efficiency, thus subsequently affecting proton pumping and ATP synthesis. Evidence from non-human organisms further demonstrates that mitogenomic variation can translate into measurable physiological phenotypes. In *Drosophila*, naturally occurring mitochondrial haplotypes have been associated with differences in metabolic rate, locomotor performance, stress resistance, and lifespan, while experimental mitonuclear combinations have revealed that the phenotypic effect of a mitochondrial variant can depend strongly on the nuclear genetic background [155,156,157]. Similar mitochondrial haplotype-dependent variation in metabolic efficiency, thermogenesis and performance has been documented in mammals and other vertebrates. In fish and ectothermic organisms, mitochondrial genetic variation can additionally influence responses to temperature and oxygen availability, providing potential links between mitogenomic diversity and environmental adaptation [158,159]. These observations indicate that mitochondrial variants are not simply molecular markers of population history, but can contribute directly to organismal phenotypes, although their effects are strongly dependent on genetic background and environmental context. Cited reports suggest that mitochondrial genetic variation may not be selectively neutral, but frequently contributes directly to functional, physiological, and behavioral phenotypes with reservations [157], which will be discussed in a separate section (Section 9) on selectivity and neutrality of molecular markers in general.

One of the most extensively reported functional consequences of mitogenomic variation involves the regulation of ROS, as mentioned earlier (Section 4). The electron leakage from mitochondrial respiratory chain complexes generates ROS, which functions both as a damaging agent (via enhancing lipid peroxidation, causing mtDNA damage, etc.) and as an efficient signaling molecule involved in regulating several cellular cascades, including stress responses, apoptosis and metabolic adaptation [160,161]. Certain mtDNA variants and haplogroups appear to modulate ROS production rates, which directly influences oxidative stress tolerance and cellular signaling cascades. The phenotypic consequences of mitogenomic variations, as noted earlier, are context-dependent. Variants that alter respiratory efficiency or ROS production may be neutral or even advantageous under particular ecological conditions, including thermal stress, hypoxia or altered nutrient availability. Comparative and experimental studies in fish, insects and other ectotherms have provided evidence that mitochondrial haplotypes can contribute to differences in thermal tolerance, hypoxia responses and energetic performance [162,163]. Such findings support a role for mitogenomic variation in local adaptation while also emphasizing that mitochondrial effect cannot be separated from nuclear genetic background or environmental conditions. In several natural populations, mitochondrial haplotypes have also shown geographically structured associations with climatic or ecological variables, suggesting that environmentally dependent selection can contribute to the maintenance of mitochondrial diversity alongside demographic processes.

Mitogenomic variation is also well known for contributing to differences in organismal aging. The mitochondrial theory of aging, often referred to as the mitochondrial free radical theory of aging, proposes that the gradual accretion of mitochondrial mutations and oxidative damage significantly contributes to functional decline over time. This may partly result from the role of mitochondria as major sources of ROS production (at complexes I and III of the electron transport chain), which can cause notable damage to cellular macromolecules [164,165,166]. A prominent role of mitochondrial ROS in aging remains debated; however, growing evidence highlights that mutations in the mitogenome that accumulate during the course of aging in multiple tissues could impair respiratory chain functions upon exceeding the threshold levels [167].

Preclinical models, particularly mtDNA-mutator mice, have provided important mechanistic evidence linking the mitochondrial genome integrity with organismal aging [168,169]. These findings complement observations in humans by demonstrating that an altered mutational burden of mitogenome can have organism-level consequences under controlled genetic and environmental conditions. An additional critical aspect of mitogenome functional is the presence of heteroplasmy, due to the coexistence of numerous mitochondrial genotypes within a cell or an organism. Since mitochondria are present in hundreds to thousands of copies per cell, their mutant variants (with mutated mitogenomes) are reported to persist at low frequencies without producing observable phenotypic effects. However, they occasionally may surpass a critical threshold level (usually ~60–80% or sometimes ~50–90% of the total mtDNA within a cell), which may compromise overall mitochondrial respiratory chain functions [170,171].

Technological advancements in ultradeep-sequencing approaches have demonstrated that low-level heteroplasmy is nearly universally present in healthy individuals, suggesting that mitochondrial populations are dynamic and continuously shaped by mutation, selection and genetic drift [172,173]. Some peculiarities concerning the role of the mitogenome and other genes in fitness will be discussed in Section 9. Mitochondrial signaling cascades interact extensively with nuclear transcriptional machinery to coordinate the efficient modulation of metabolic networks, emphasizing the importance of combined genomic reprogramming [174]. Evidence across animals, plants, and other eukaryotes indicates that the functional consequences of mitogenomic variation extend far beyond human disease [175]. In natural populations, mitochondrial variants can influence metabolic efficiency, thermal and hypoxic tolerance, stress resistance, reproductive performance and lifespan, thereby providing potential substrates for natural selection [157,158,159,160,161,162,163,164,165,166,167,168,169,170,171,172,173,174,175,176]. However, the direction and magnitude of these effects may vary among species and depend on nuclear background, environmental conditions, heteroplasmy and demographic history. Thus, mitogenomic variation may represent both a source of physiological constraint and a substrate for adaptive [177] and non-adaptive or stochastic evolution. Altogether, these observations establish mitogenomes as dynamic determinants of cellular physiology, organismal performance and disease susceptibility rather than passive repositories of bioenergetic genes. Across evolutionary timescales, mitogenomic variation can influence metabolic efficiency, stress responses and life-history traits, while in humans similar mechanisms may manifest as mitochondrial disorders or contribute to complex diseases [178]. These functional consequences arise through interactions among OXPHOS efficiency, ROS signaling, heteroplasmy, cellular regulation, nuclear genetic background and environmental context, providing a mechanistic basis for mitonuclear coevolution.

## 6. Mitonuclear Interactions and Coevolution

The dynamic functioning of mitochondria depends explicitly on intricate genetic integration between the mitogenomes and the nuclear genomes. While the former encode 13 PCGs and a few others, the majority of proteins (~99%) are nuclear-encoded, like in mammals and most eukaryotes, and subsequently imported into the individual organelle after cytosolic translation [179]. This inter-genomic division of functional loads suits the generation of a cellular environment wherein mitochondrial and nuclear genes may evolve in a coordinated manner. As a result, this mechanism allows functioning of oxidative phosphorylation and maintenance of metabolic homeostasis. Accordingly, mitonuclear coevolution has emerged as a significant frontier in the field of mitochondrial biology, connecting molecular evolution with organismal fitness, speciation and disease severity [180]. The need for such intergenomic and organismal synchronized evolution arises predominantly from the structural composition of mitochondrial respiratory chain complexes and their integration in eukaryotic cellular organization. Key components of respiratory chain complexes (I, III, IV and V) contain both mtDNA- and nDNA-encoded subunits that assemble into multi-subunit macromolecular structures embedded within the inner mitochondrial membrane [31]. Comparative genomic analyses highlight signatures of such compensatory evolution across diverse taxa, particularly in genes encoding respiratory chain complexes [20,181]. The molecular basis of mitonuclear coadaptation is particularly evident at physical interfaces within OXPHOS complexes. Mitogenome-encoded subunits form the hydrophobic core and catalytic or electron-transfer components of respiratory complexes, whereas numerous nuclear-encoded subunits contribute to ultrastructural stabilization, their assembly, precise regulation, and required catalytic function [20]. Consequently, amino-acid substitutions in mitogenome-encoded proteins can alter residue contacts, local protein conformation, membrane interactions or electron-transfer properties, and thereby modify the compatibility of interacting nuclear-encoded partners [182]. Nuclear compensation can subsequently occur through amino-acid substitutions, altered protein abundance or changes in assembly and regulatory factors that restore structural and functional compatibility. Such compensatory evolution need not involve direct residue-for-residue matching: changes at one protein interface may be accommodated by alterations elsewhere in the interacting complex or by modifications in protein expression and mitochondrial quality-control pathways [20,180,181]. Further insights into these matters are discussed in Section 9.

Evidence for mitonuclear coadaptation has been especially strong in hybridization studies. Cytonuclear incompatibility can arise when mitochondrial and nuclear genetic combinations that evolved in separate populations or species are brought together and lack sufficient compensatory interactions to maintain mitochondrial function [183]. At the molecular level, incompatibility can result from altered physical interactions between mitogenome- and nuclear-encoded OXPHOS subunits, impaired assembly or stability of respiratory complexes, disrupted electron transfer or proton translocation, and inappropriate regulation of mitochondrial gene expression [184]. These defects can increase respiratory inefficiency and ROS production while activating mitochondrial stress and proteostasis responses. In hybrids, such molecular defects may manifest as reduced ATP production, impaired development, altered fertility, or reduced survival [185]. Importantly, incompatibility is not necessarily caused by a single mtDNA mutation: it can emerge from multi-locus interactions in which several mitochondrial and nuclear variants collectively determine respiratory-chain performance. Thus, cytonuclear incompatibility provides a mechanistic route through which mitonuclear divergence can contribute to hybrid dysfunction and under appropriate evolutionary conditions reproductive isolation [94,186,187].

Moreover, mitonuclear discordance represents incongruence between mitochondrial and nuclear evolutionary histories or patterns of genetic differentiation and should be distinguished from functional cytonuclear incompatibility. Such discordance could originate due to mitochondrial introgression following hybridization, incomplete lineage sorting, differences in Ne, sex-biased dispersal, selective sweeps and demographic history [188]. For example, mitochondrial haplotypes may cross population or species boundaries through hybridization and concomitant backcrossing, whereas most of the nDNA remains associated with the recipient lineage, producing mitochondrial and nuclear genealogies that support different evolutionary histories [189]. Importantly, such discordance does not necessarily indicate impaired mitochondrial dynamics. However, introgressed mitogenomes may remain compatible with the recipient nuclear background, whereas stronger divergence at interacting mitochondrial and nuclear loci can expose functional incompatibilities. Thus, mitonuclear discordance may represent an evolutionary intermediate between independent genomic histories and functional cytonuclear mismatch, with its phenotypic and evolutionary consequences shaped by the extent of molecular coadaptation and the prevailing ecological and demographic context [190].

Experimental systems such as *Tigriopus californicus*, *Nasonia* and *Drosophila* have been particularly informative because controlled mitochondrial–nuclear combinations allow the fitness consequences of cytonuclear mismatch to be separated from broader population-level effects. These studies demonstrate that mitochondrial genotype can interact strongly with nuclear background to influence respiratory activity, development, stress tolerance and reproductive performance [187,188,189,190,191,192,193,194,195,196]. However, the occurrence of mitonuclear genealogical discordance following hybridization does not by itself establish functional incompatibility, because introgressed mitochondrial haplotypes may persist without detectable fitness effects when functional compatibility with the recipient nuclear background is maintained [197]. Such findings support a model in which mitonuclear compatibility is a property of specific genomic combinations rather than an intrinsic characteristic of an individual mitochondrial haplotype. However, data supporting such claims have remained limited since the 1980s [94,175,193,194,195,196]. Mitonuclear interactions are also known to influence adaptation during environmental challenges. For instance, changes in climatic conditions, oxygen availability, or bioenergetic demand may themselves select for mitochondrial variants that optimize electron transport efficiency under new environmental stressors [198].

Since these mitochondrial variants interact with nuclear-encoded proteins, adaptive evolution may therefore encompass coordinated substitutions in both mtDNA and nDNA genomes. For examples, investigations of high-altitude vertebrates have identified significant mitochondrial and nuclear changes related to heightened oxidative phosphorylation under hypoxic conditions. Such adaptations include a higher evolutionary rate in mitochondrial PCGs compared to low-altitude species, potentially optimizing the oxygen utilization [199]. Similar patterns have also been observed in insects and marine organisms adapting to thermal gradients [199,200,201,202]. A recent study showed higher nucleotide diversity and evolutionary rate in an alpine group compared to a lowland group, wherein mitogenome PCGs (ATP6, ATP8, COX1, COX3 and CYTB) showed seven potentially positive selection sites, whereas other genes (ATP6, ATP8, COX3 and ND1) were also found to be significantly associated with temperature-related environmental factors. These findings offer deeper insights into adaptive mechanisms of parasitoids in response to the alpine environment and subsequently highlight how such species might respond to future ambient-temperature shifts [203,204].

By modulating evolutionary dynamics, mitonuclear interactions have important implications for human health and any disruption in such coordinated network could cause mitochondrial failure and commencement of disease. Classical mitochondrial disorders (such as mitochondrial encephalomyopathy with lactic acidosis and stroke-like episodes and myoclonic epilepsy with ragged red fibers) are known to arise from pathogenic mutations in either the mitogenome or nuclear genes encoding mitochondrial assembly, electron-transfer efficiency, ROS homeostasis and mitochondrial stress signaling. Consequently, pathogenic mtDNA variants may produce markedly different phenotypes de-pending on the nuclear genetic background, while nuclear variants affecting mitochondrial proteins can modify the penetrance or severity of mtDNA-associated diseases [204,205,206]. In humans, disruption of mitonuclear compatibility can affect the respiratory complex and is particularly relevant in the context of mitochondrial replacement therapies, wherein nuclear genomes are paired with donor mitochondria to prevent the transmission of pathogenic mitogenome variants [207]. Together, these observations support a model in which mitonuclear coevolution operates through reciprocal molecular constraints imposed by physically interacting gene products and shared mitochondrial processes [20,208]. Selection on one genome can alter the functional landscape experienced by variants in the other, while compensatory substitutions, changes in gene expression, and mitochondrial quality-control mechanisms can maintain biochemical compatibility [209]. Conversely, disruption of these relationships through mutation, hybridization, introgression or environmental change can expose cytonuclear incompatibilities and im-pair respiratory function [210]. Mitonuclear coevolution therefore provides a mechanistic bridge connecting molecular sequence divergence with physiological variation, reproductive isolation, adaptation and disease susceptibility. Generally, that framework can be useful for construction of a molecular-and-causal network, which could help in estimation of classical general fitness (W_G_) solving in the equation W_G_ = W_F_ + W_V_ (where W_F_ + W_V_ represent fertility and viability components, respectively).

## 7. Phylogenetic Utility of Mitogenomes

The small size, high copy number, maternal inheritance, lack of recombination, and rapid evolutionary rate, make mitogenomes particularly useful for field research. These noteworthy characteristics have made mtDNA sequences particularly valuable for reconstructing species-level phylogenetic relationships, inferring population history, and identification of populations or phylogeography, etc. [80,211,212]. Since the initial applications of mitochondrial markers in molecular systematics, mtDNA has played a significant role in shaping modern phylogeography and advancing our understanding evolutionary genetics [213]. One of the primary advantages of employing mtDNA markers for evolutionary inference is their predominantly uniparental inheritance and lack of recombination in most animals. This facilitates the generation of genealogies that are easier to reconstruct than those at the recombining nuclear loci (due to lack of the confounding effects of genetic shuffling that occur in nDNA [213]). This often facilitate lineage sorting, thus expediting resolution of recent divergence events [1,12], with certain reservations mentioned above and considered in more detail by Avise [213]. Additionally, the relatively higher mutation rate observed in animals’ mtDNA further augments its utility for investigating population-level variation and demographic history, facilitating the tracking of migration patterns, population expansions, and genetic bottlenecks across diverse taxa [214,215].

Comprehensive sequencing approaches have considerably expanded the phylogenetic information accessible from mitogenomes. Mitogenomic datasets from higher eukaryotes encompass not only 13 protein-coding genes but also two rRNAs, 22 tRNAs, and CRs, which collectively provide extensive phylogenetic signals capable of resolving relationships at diverse taxonomic levels, from population genetics to higher-order systematics [35,71,216]. Subsequently, mitogenomes have also been intensively employed to reconstruct deep evolutionary relationships across vertebrates, invertebrates, and some microbial eukaryotes, e.g., as recently demonstrated in studies of the human microbiome [217]. In addition to mitogenome sequence information, gene-order rearrangements themselves can also serve as phylogenetic characters, offering independent evidence for evolutionary relationships among lineages [218,219,220]. Despite these advantages, reliance on mitogenomes also has several limitations. Since mtDNA represents a single non-recombining locus, its genealogy may not precisely reflect the evolutionary history of entire nuclear genome or the species tree. This is because mtDNA is particularly susceptible to processes that deviate from species branching history, such as incomplete lineage sorting, introgression and selective sweeps, thereby producing discordance amongst mitochondrial and nuclear phylogenies. In some cases, mitochondrial introgression between species can obscure true species boundaries or generate misleading signals of evolutionary relationships [213,221,222,223,224].

Selection acting on mitochondrial genes may also bias phylogenetic inference, as the mitogenome does not always represent the neutral markers often assumed in molecular systematics, but instead represents a complex entity [35,71,212]. Although mtDNA is widely used for investigating patterns of recent evolutionary history, by consequent demonstrations of positive and purifying selection, there is frequently neither (i) precise documentation for the fact that natural selection indeed acts on these molecular markers, nor (ii) adherence to the expectations of evolutionary models. For instance, whereas metabolic adaptations may lead to divergent mitochondrial loci, which concomitantly yield conflicting phylogenetic trees and generate inaccuracies in identification of true species relationships [35,71,225,226,227]. Since mtDNA encodes for crucial components of the mitochondrial protein subunits of the OXPHOS complexes, it is strongly influenced by both adaptive evolution (driving the fixation of advantageous mutations in specific environments) and purifying selection (involved in removing deleterious mutations to maintain normal functions). This multifaceted selection pattern in combination with its distinctive features (high substitution rates, lack of recombination, and maternal inheritance) results in lineage-specific rate variation in mitochondrial populations. Such deviations from neutral expectations can substantially confound molecular clock estimates and phylogenetic reconstruction if not appropriately accounted for [35,71,198,212,228].

These effects can be accommodated by explicitly modeling heterogeneity in mitochondrial evolutionary rates and selective constraints. Phylogenetic analyses can employ partitioned substitution models that allow different evolutionary processes among genes or codon positions, whereas relaxed molecular-clock models can accommodate lineage-specific variation in substitution rates, rather than imposing a single rate across all branches [229,230]. Tests of selection based on Dn/Ds patterns can further identify genes or lineages showing departures from predominantly purifying evolution, thereby informing the choice of evolutionary models [231]. Where demographic history, introgression, or incomplete lineage sorting is plausible, mitochondrial phylogenies should additionally be evaluated against independent nuclear or multilocus datasets [232]. Calibrating mitochondrial clocks using multiple reliable fossil, biogeographic or secondary calibrations, and conducting sensitivity analyses under alternative rate and clock models, can further reduce the influence of model-dependent assumptions. Thus, rather than treating mtDNA as a universally clock-like marker, incorporating lineage-, gene-, and codon-specific rate variation could provide a more robust framework for phylogenetic and divergence-time inference [233]. The large-scale accessibility of nuclear genomic data accelerated by next-generation sequencing strategies has encouraged a shift towards employing an integrative phylogenomics approach, wherein mitochondrial and nuclear markers are analyzed together to reconstruct evolutionary history framework while addressing the limitations inherent in using either marker type alone. In such technological approach, mitogenomic data remain a valuable toolset due to its their high resolution for recent divergences and capability to offer autonomous and multiple sets of evolutionary patterns. When combined with genome-wide nuclear datasets, mitogenomes can provide robust phylogenetic hypotheses to identify complex processes such as introgression [234,235].

**Figure 3:**
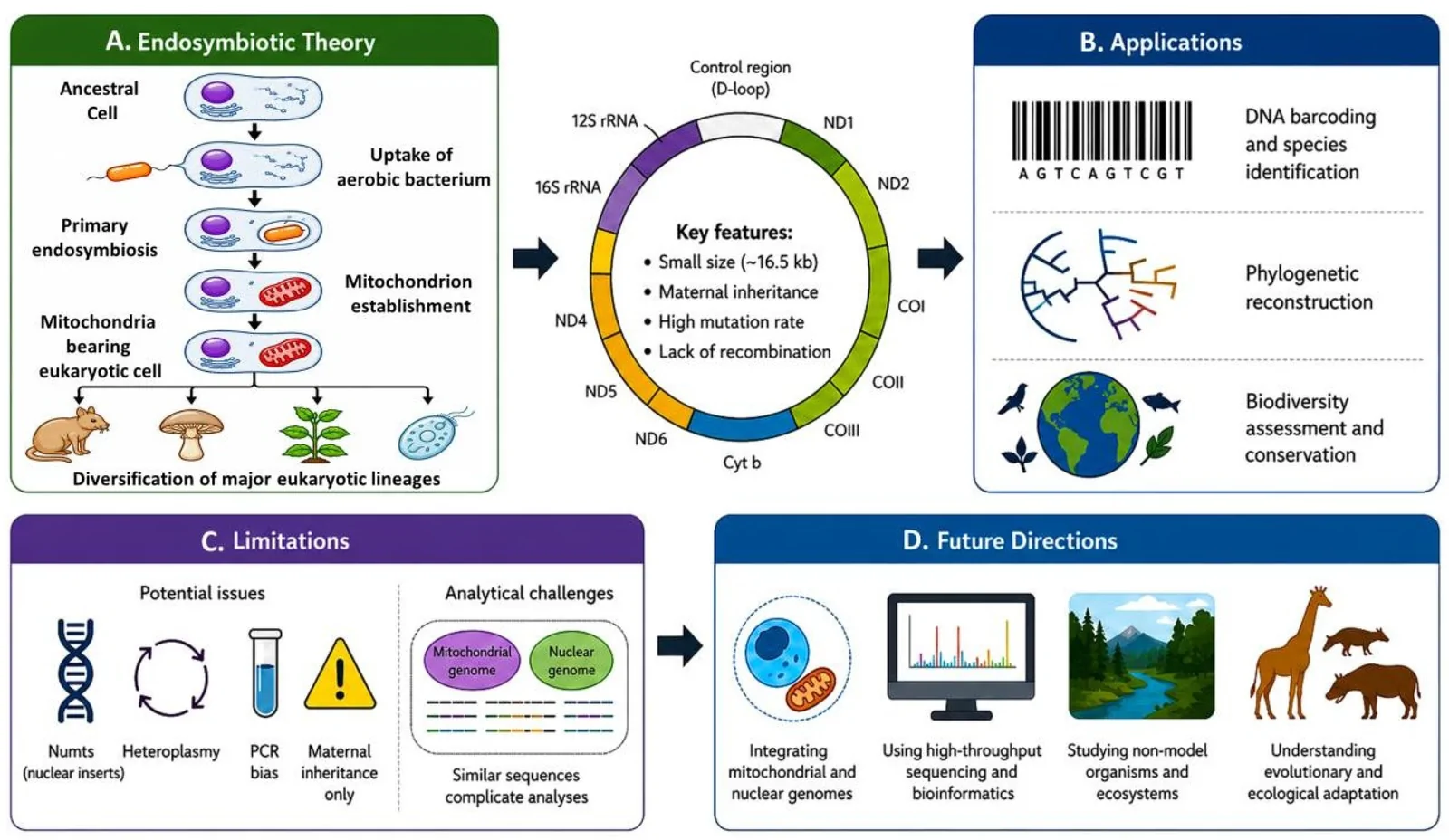
Mitochondrial genomics in species identification and evolutionary research. Author’s overview of the conceptual and analytical framework of mtDNA applications, wherein the endosymbiotic origin of mitochondria highlights the evolutionary transition from ancestral eukaryotic cells to mitochondria-bearing cells (Panel (**A**)). The central panel depicts circular mtDNA, emphasizing key features such as compact size (~16.5 kb), maternal inheritance, elevated mutation rate, and absence of recombination. Panel (**B**) summarizes major applications, including DNA barcoding for species identification, phylogenetic reconstruction and biodiversity assessment. Panel (**C**) depicts key limitations, such as nuclear mitochondrial insertions, heteroplasmy, PCR bias and analytical challenges arising from sequence similarity between mtDNA and nuclear genomes. Future directions (Panel (**D**)) focus on integrative multi-genome approaches, high-throughput sequencing with advanced bioinformatics, and expanding studies across understudied taxa and ecosystems to improve our understanding of evolutionary dynamics and ecological adaptation.

Recent technological advancements in sequencing methodologies have also expanded the use of mtDNA information in several other scientific domains, such as in environmental DNA (eDNA) analysis, ancient DNA studies, and biodiversity monitoring. Since mitochondrial genes occur usually at high copy number and often persist in degraded samples. They are particularly useful for identifying species presence in environmental samples or reconstructing evolutionary histories from ancient remains [236]. Altogether, mitogenomes remain indispensable tools in the field of evolutionary genetics. Modern technological approaches increasingly emphasize the integration of mitogenomic information with nuclear genomic data and ecological findings. This holistic perspective allows researchers to exploit the strengths of mitochondrial markers while mitigating their limitations, offering deeper insight into the evolutionary processes shaping biodiversity (Figure 3).

## 8. Technological Advances and Future Directions

Recent technological advances are rapidly transforming the study of mitochondrial genomes, facilitating improved precision in characterization of genome architecture, population dynamics, molecular–genetic detailing of metabolic chains, and functional integration of mitogenomes with entire cellular systems. Such cutting-edge developments are reshaping long-standing concepts in mitochondrial genetics and offering novel approaches, summarized in eight key areas for simplicity (Table 2), with promising translational applications [5] and implications for cell biology in general. Recently, the successful establishment of long-read sequencing platforms developed by Pacific Biosciences and Oxford Nanopore Technologies has significantly enhanced the resolution by which mitogenomes can precisely be assembled and analyzed. Unlike short-read sequencing approaches, the long-read platforms generate long contiguous reads in several kb in length, facilitating the precise reconstruction of complex mitochondrial structures including repeat regions, complex structural rearrangements, and multipartite genome configurations [237,238]. These advanced technological approaches have demonstrated their effectiveness in resolving mitogenomes of plants, protists, and other metazoans, wherein repeated sequences and recombination-mediated rearrangements have previously hindered assembly via short-read methods. For example, long-read sequencing of genomic DNA from adult *Schistosoma haematobium* enabled the assembly of complete mitochondrial non-coding regions that had previously remained unresolved. The recovery of contiguous reads spanning approximately 18.5 kb enabled the resolution of these previously unresolved non-coding regions, illustrating how long-read data can complement conventional sequencing approaches for structurally complex mitogenomes [239].

In another study, involving three species of sea chubs, it was demonstrated that perfect (complete and fully accurate) or quasi-perfect (complete, but containing single or very few errors) mitogenomes can be assembled at high (>25×) and low (3–5×), but not at very low (1×, genome skimming) sequencing depths using long-reads [238].

Such long-read sequencing approaches have also enhanced the detection of structural heteroplasmy, highlighting the possibility that mitochondrial populations within cells may consist of multiple genome conformations rather than existing as a single canonical structure. This challenges the classical view of mitochondria as genetically uniform entities and suggests that mitochondrialtheir genome organization may be more complex than previously reported [255]. In addition, advances in targeted enrichment and single-molecule sequencing have further facilitated high-resolution mapping of mtDNA mutations and detection of copy-number variations in individual cells, offering unique insights into mitochondrial population genetics and somatic mosaicism at the cellular level [240,241]. Recently, an individual mitochondrial genome sequencing (iMiGseq) approach for full-length mtDNA was developed for ultrasensitive variant detection, complete haplotyping, and unbiased evaluation of heteroplasmy levels at the individual mtDNA molecule level. [242]. This is not only beneficial in elucidating the mitochondrial etiology of diseases, but also in investigating the safety of several mtDNA editing approaches [242]. An ultradeep approach of the amplicon-based sequencing library was also designed that could cover the entire mitochondrial genome via high-depth mtDNA sequencing for detecting mutations in mtDNA linked to risk, progression, and treatment response of head-and-neck squamous cell carcinoma (HNSCC). This approach rapidly sequenced mtDNA mutations in 28 HNSCCs matched with lymph node metastasis, surgical margins, and bodily fluids, including multiregional sequencing, on 14 primary tumors. This offered a comprehensive overview of mitochondrial heterogeneity, which may be useful for detecting low-frequency tumor-associated mtDNA mutations in lymph nodes and sputum and serum specimens from cancer patients [243].

Despite these advances, mitochondrial sequencing and assembly remain subject to important technical and computational limitations. Long-read platforms provide improved resolution of repetitive and structurally complex regions, but individual platforms differ in raw-read accuracy, error profiles and susceptibility to homopolymer-associated or context-dependent errors [256,257]. Short-read approaches generally provide high base-level accuracy, but may inadequately resolve repeats, large deletions, heteroplasmic structural variants, and complex genome configurations [258]. Conversely, increased read length does not by itself guarantee accurate assembly, particularly when mitochondrial molecules occur as heterogeneous populations or when nuclear mitochondrial DNA segments (NUMTs) are co-amplified [259,260]. Variant detection is similarly sensitive to sequencing depth, library preparation, alignment strategy, error correction, and variant-calling thresholds. These factors are particularly important for low-frequency heteroplasmy, where technical errors may overlap with the allele-frequency range of biologically meaningful variants. Interpretation of heteroplasmy remains especially challenging because mitochondrial genomes within the same cell, tissue, or individual may exist as a dynamic mixture of sequence and structural variants. Low-frequency variants may represent genuine biological heteroplasmy, but can also arise from polymerase errors, sequencing artefacts, PCR amplification bias, index misassignment or alignment errors [261,262]. Conversely, highly divergent mitochondrial haplotypes or structural variants may be under-represented by conventional amplification-based approaches. Heteroplasmy measurements are therefore dependent not only on biological state but also on experimental design and analytical thresholds [263]. Tissue specificity, mtDNA copy number, age, clonal expansion, and selective processes further complicate comparisons between individual or longitudinal samples. Robust interpretation consequently requires adequate sequencing depth, technical replication, orthogonal validation, and single-molecule or independent library approaches where possible [242,264].

Besides structural genomics, the integration of mitochondrial data into system-level analyses has recently demonstrated how mitochondrial variation influences larger cellular signaling. Combined analysis of mitochondrial and nuclear genomic data is also indispensable for detecting mitonuclear discordance. Comparative phylogenomics, genome-wide population analyses, and explicit tests of introgression can distinguish genuine cytonuclear co-divergence from mitochondrial histories shaped by incomplete lineage sorting, demographic processes or selective sweeps [264,265]. Future analytical approaches should therefore integrate mitogenomic and nuclear markers rather than treating mitochondrial genealogies as independent entities for species history. Multi-omics approaches combining genomics, transcriptomics, proteomics, and metabolomics have revealed extensive “cross talk” between mitochondrial function and nuclear gene regulation [266,267]. For instance, compromised mitochondrial dynamics have been demonstrated for activation of retrograde signaling cascades involved in altering nuclear transcriptional workflows, thus modulating metabolic responses across cellular compartments [268]. Recent studies employing single-cell transcriptomics and spatial omics technologies have further demonstrated that mitochondrial gene expression differs significantly across tissues and sometimes during the different developmental stages. Such observations reflect the dominant role of mitochondria in regulating energy metabolism, redox homeostasis and apoptosis. Therefore, integrating these datasets with quantitative measurements of mitochondrial respiration and metabolite flux could assist in establishing predictive models linking mitochondrial genetic variation with physiological outcomes [245,246].

Despite rapid progress, several fundamental questions about mitochondrial biology still remain unresolved. One of the key challenges is in understanding how mitonuclear coevolution operates across evolutionary timescales and ecological contexts. Evidence for mitonuclear incompatibilities is accumulating, given the high observing mutation rate of mtDNA and the tight, co-evolved requirement for strong coevolutionary dependence of mitochondrial function on interactions between mitochondrial and nuclear-encoded proteins. Hence, the molecular mechanisms by which nuclear genomes compensate for mitochondrial mutations remain incompletely characterized. Determining these mechanisms will require integrative approaches combining structural biology, evolutionary genomics, and functional assays [94,252]. Equally important is the need to clarify the role of mitochondrial heteroplasmy in the development of complex diseases and the aging process. While advances in sequencing have revealed widespread low-level heteroplasmy, the extent to which these variants influence cellular function and disease susceptibility remains an active area of investigation [269,270]. Longitudinal studies tracking mitochondrial mutation dynamics across tissues and lifespans will be essential for clarifying these relationships. Translational opportunities are also expanding. Emerging therapeutic strategies, including mitochondrial gene editing approaches (mitoTALENs, DddA-derived base editors), targeted nucleases (mtZFNs), and mitochondrial replacement techniques (e.g., spindle transfer, pronuclear transfer), aim to prevent the transmission of pathogenic mtDNA mutations or restore mitochondrial function in affected tissues [271,272].

Advances in machine learning approaches have also been applied to predict genetic relatedness using human mtDNA hypervariable region I sequences. Overall, the results obtained using WEKA (version 3.8.6) and Python (version 3.11) showed that different machine learning models achieved the highest accuracy for the Caucasian population, followed by the African population, with the lowest accuracy obtained for the Asian population [253]. The authors suggested that the higher genetic diversity of the African populations may make it more difficult to classify them, and pointed to a higher degree of similarity in the Caucasian race than the African and Asian races [253,254]. The clinical translational success of such technological platforms will depend on a deeper understanding of mitonuclear compatibility and mitochondrial population dynamics. Altogether, advances in sequencing technologies, systems biology, and translational medicine are still redefining the frontiers of mitochondrial research. By integrating structural genomics with functional and evolutionary perspectives, future investigations are believed to further uncover how mitogenomes shape biological diversity, promote environmental adaptation, and hence contribute in sustaining human health.

## 9. Selectivity and Neutrality of Variability at Molecular Markers

Earlier it was briefly discussed how mitogenome properties could contribute to local adaptation [140,273] and large differences between Dn and Ds substitution rates in many PCGs or other elements of mitogenomes in a variety of Eukaryotic linages. Claims on local adaptation in studies such as in the above cited reports [140,273], are experimental investigations on murine cell culture and on laboratory organisms such as Drosophila. Both findings cited above are thorough investigations, but they offer limited valuable evidence in terms of selection in natural populations at the level of molecular markers, including the mitogenome. They only demonstrate that selective neutrality cannot fully account for the findings in these investigations and that a variety of factors, including selective forces, play roles in the development and population dynamics of organisms [32,71,274]. Recent findings on one of the most abundant bivalves, the mussel family Mytilidae, have provided insights into mitogenome structure, evolution, and the effects of natural selection on molecular polymorphisms in nature [35,71,274]. Available data have emphasized the complexity of the phenomenon and in many cases the lack of precise explanations for the selective effects observed at molecular markers in natural populations [35,71,275,276]. Nevertheless, mitogenomic variations are also known for direct influence on organismal traits, including fertility, stress tolerance, and immune responses, as noted above and discussed further at the end of this section. For instance, in both insects and vertebrates, mtDNA haplotypes can contribute to local adaptation, with haplotype-specific differences affecting reproductive success through mitonuclear interactions and influencing thermal performance. Thus, mtDNA variation can play an important role in shaping contemporary local adaptation [140,273,277].

The interpretation of mitogenome variation has long been influenced by the debate between neutrality and selection in molecular evolution. Traditionally, mitogenome markers were assumed to evolve largely under the principles of neutral theory [278,279,280], whereby most polymorphisms arise through mutations and are subsequently shaped by genetic drift, rather than adaptive processes [80,281]. This assumption underpins the widespread use of mitochondrial markers in phylogeography, population genetics, and species identification. However, growing evidence suggests that many mitochondrial variants are subject to varying degrees of purifying, positive, and balancing selection, challenging the view of strict neutrality [80,282]. Purifying selection remains the predominant force acting on mitochondrial protein-coding genes given their essential roles in OXPHOS. Consequently, deleterious mutations are frequently removed from populations, contributing to the high conservation of core mitochondrial functions [283,284]. Conversely, adaptive evolution has been documented in diverse taxa inhabiting extreme environments, including high-altitude vertebrates, polar fishes, and migratory birds, where mitochondrial variants influence metabolic efficiency and environmental fitness [199]. At the same time, demographic processes, reduced Ne, and uniparental inheritance can facilitate the fixation of mildly deleterious mutations through genetic drift, particularly in small or bottlenecked populations [135,285].

These observations indicate that mitochondrial markers exist along a continuum between neutrality and selection rather than representing purely neutral genetic systems. Importantly, selective pressures may vary among mitochondrial genes, taxa, and ecological contexts, influencing rates of sequence evolution and patterns of genetic diversity [286,287]. Recognizing the interplay between selection and neutrality is therefore essential for accurate interpretation of mitogenomic data, particularly in studies of adaptation, phylogenetic inference, population structure, and mitonuclear coevolution [208,287,288]. Such considerations provide a more nuanced framework for understanding the evolutionary significance of mitochondrial genome variability across biological systems.

## 10. Conclusions

Mitogenomes are uniquely repositioned at the intersection of evolutionary genetics, cellular physiology, and human well-being. Although traditionally, mitogenomes have been considered compact remnants of an ancestral endosymbiont that are principally responsible for ATP generation, present-day findings reveal them as dynamic genetic systems whose morphological architecture, inheritance patterns, and diverse interactions with nuclear genes modulates biological processes across multiple levels of organization. The emergent recognition of mitochondrial functional complexity has necessarily reshaped how mitogenomes are investigated and interpreted. Across eukaryotes, mitogenomes demonstrate remarkable diversity in size, structure, and evolutionary patterns. Comparative analyses validate that mitogenome architecture reflects an equilibrium between functional constraints imposed by OXPHOS functioning, and lineage-specific evolutionary pressures, including mutation rate, recombination, population size, and life-history traits. Concurrently, such factors promote the generation of a spectrum of genomic configurations, ranging from streamlined circular genomes (which are typically observed in most animals) to highly expanded and re-combination-prone mitogenomes (found in plants and certain protists). Such genetic diversity challenges classically established assumptions of mitochondrial uniformity and accentuates the significance of employing comparative genomics for understanding mitochondrial evolution.

Moreover, equally imperative is the observation that mitogenomes cannot be studied in isolation from the nuclear genome. The indispensable functions of mitochondria are predominantly dependent on highly synchronized network of genes distributed across both the genomes. Such interdependence facilitates the mitonuclear coevolution phenomenon, involved in maintaining compatibility between mitochondrial and nuclear-encoded components of the respiratory chain. Any disruption of this synchronized phenomenon—whether via hybridization, higher mutation rates, or environmental stress factors—could significantly impair cellular bioenergetics and concomitantly influence organismal fitness. Consequently, mitonuclear cross talk offers a platform for linking molecular evolution with physiological functioning, ecological adaptation, and speciation. Technological advancements in high-throughput sequencing approaches have transformed our understanding of progression in mitochondrial diseases and their subsequent variation. The widespread existence of mitochondrial heteroplasmy, accretion of somatic mtDNA mutations (with age), and compromised mitochondrial dynamics in numerous complex disorders highlights the fundamental role of mitogenomes in sustaining human health. Subsequently, recently developed therapeutic approaches—such as mitochondrial replacement technologies and targeted editing of mitogenomes—offer promising results in addressing inherited mitochondrial disorders. Such technological developments demonstrate how data insights from findings in evolutionary genetics can be transformed into clinical innovations. From a futuristic point of view, numerous key challenges and technological advancements will reshape the next phase of mitochondrial research. For example, integrating mitochondrial genomics with systems-level approaches will be indispensable for deciphering how variation in mitogenome orchestrates cellular signaling network and organismal phenotypes. Importantly, progress in long-read sequencing technologies, single-cell genomics, and multi-omics integration is highly expected for further strengthening the concept of dynamic existence of mitochondrial populations within cells and across the tissues. Simultaneously, resolving unanswered queries concerning mitonuclear compatibility, heteroplasmy dynamics, and the evolutionary drivers of mitochondrial diversity could require assimilating interdisciplinary methodological approaches that could bridge molecular biology, evolutionary theory, and clinical research.

In summary, mitogenomes are far more than just bioenergetic entities. Rather, they should be considered dynamic evolutionary structures whose synchronized cross talk with nuclear genomes and cellular microenvironments influences the diversity, adaptability, and health of living organisms. This warrants the sustained integration of evolutionary, functional, and translational approaches in uncovering the full biological significance of mitogenomes in the near future.

## Figures and Tables

**Table 1:** Mitogenomic variations and their functional consequences in physiology and disease.

Mitogenomic Characteristic Feature	Molecular Mechanism	Physiological Consequences	Associated Diseases/ Phenotypes	Translational Implications	References
**Heteroplasmy**	Coexistence of mutant and wild-type mtDNA	Threshold-dependent impairment of oxidative phosphorylation	MELAS, LHON, MERRF	Target for mitochondrial replacement therapies	[146,147]
**Mitogenome mutation rate**	Accumulation of substitutions in mitochondrial genes	Altered respiratory chain efficiency	Aging-related decline, neurodegeneration	Biomarker for disease progression	[148,149]
**Mitonuclear incompatibility**	Mismatch between mitochondrial and nuclear gene products	Impaired OXPHOS complex assembly	Metabolic disorders, incompatibilities in hybrid individuals	Personalized mitochondrial medicine	[21,150]
**Mitogenome deletions**	Excision of mitochondrial genomic segments	Reduced ATP production	Human Kearns–Sayre syndrome, mitochondrial myopathies	Gene editing and therapeutic targeting	[151,152]
**Adaptive mitogenome variation**	Purifying and positive selection on mitochondrial proteins	Environmental metabolic adaptation	High-altitude adaptation, thermogenesis	Evolution-informed therapeutic insights	[153,154]

Note: Data in the table compiled by the authors from the references cited in right column.

**Table 2:** Emergent technological and analytical advancements in mitogenome research.

No	Technology/ Approach	Working Principle	Key Applications in Mitogenome Research	Major Advantages	Present Limitations	References
**1**	Long-read sequencing (e.g., Pacific Biosciences, Oxford Nanopore Technologies)	Single-molecule sequencing generating long contiguous reads	Assembly of complete mitochondrial genomes; detection of structural rearrangements, resolution of repetitive regions	Enables accurate assembly of complex mitogenomes, reveals structural heteroplasmy and genome isoforms	Higher error rates relative to short-read platforms, computation al correction often required	[237,238,239]
**2**	Single-cell mitochondrial genomics	Sequencing mitochondrial DNA from individual cells	Mapping heteroplasmy dynamics, studying mitochondrial mutation accumulation across tissues	Resolves cell-to-cell variation in mtDNA, reveals clonal expansion of mitochondrial mutations	Limited DNA input, amplification bias can affect variant detection	[240,241,242]
**3**	High-depth mtDNA sequencing	Ultra-deep sequencing of mitochondrial genomes	Detection of low-frequency heteroplasmic variants; somatic mutation profiling	High sensitivity for rare mtDNA variants, useful for clinical diagnostics	Requires high sequencing coverage and careful error filtering	[243,244]
**4**	Multi-omics integration	Integration of genomics, transcriptomic s, proteomics, and metabolomics datasets	Systems-level analysis of mitochondrial function and regulation	Enables understanding of mitochondrial signaling networks and metabolic integration	Complex data integration, requires advanced computation al methods	[245,246,247]
**5**	Spatial transcriptomics	Mapping gene expression within tissue architecture	Investigating tissue-specific mitochondrial gene expression and metabolic specialization	Maintain spatial context of mitochondrial activity	Limited resolution for mitochondrial transcripts in some platforms	[24,248]
**6**	Environmental DNA (eDNA) metabarcoding	Sequencing mitochondrial markers from environmental samples	Biodiversity monitoring, detection of rare or cryptic species	Sensitive species detection without direct sampling	PCR biases, incomplete reference databases	[249,250,251]
**7**	Ancient DNA sequencing	Recovery of degraded DNA from archaeological samples	Reconstruction of evolutionary history and population dynamics	Enables study of extinct or ancient populations	DNA fragmentation and contamination risks	[94,252]
**8**	Machine learning in evolutionary genomics	Computational models detecting patterns in large genomic datasets	Phylogenetic inference, mutation prediction, and evolutionary modelling	Handles large genomic datasets, identifies hidden evolutionary patterns	Requires extensive training datasets and validation	[253,254]

## Data Availability

No new data were created or analyzed in this study. Data sharing is not applicable to this article.

## Abbreviations

Dn/Ds	substitution rates for non-synonymous and synonymous mutations (substitutions)
nDNA	nuclear DNA
mtDNA	mitochondrial DNA
PCGs	protein-coding genes
